# Intravenous theophylline is the most effective intervention to prolong EEG seizure duration in patients undergoing electroconvulsive therapy

**DOI:** 10.1186/s12871-017-0412-5

**Published:** 2017-08-29

**Authors:** Alexander Tzabazis, Michaela E. Wiernik, Jan Wielopolski, Wolfgang Sperling, Harald Ihmsen, Hubert J. Schmitt, Tino Münster

**Affiliations:** 1 Department of Anesthesiology, Perioperative and Pain Medicine, Stanford University, School of Medicine, 300 Pasteur Dr, Stanford, CA 94305 USA; 2 Department of Anesthesiology, University Hospital of Erlangen, Krankenhausstrasse 12, 91054 Erlangen, Germany; 3 Department of Psychiatry and Psychotherapy, University Hospital Zürich, Culmannstrasse 8, 8091 Zürich, Switzerland; 4 Department of Psychiatry, University Hospital of Erlangen, Schwabachanlage 6, 91054 Erlangen, Germany

**Keywords:** ECT, Seizure duration, Theophylline, Remifentanil, (S-)ketamine, Barbiturate, Thiopental, Etomidate, Abbreviations., ECT Electroconvulsive therapy, EEG Electroencephalogram., US United States.

## Abstract

**Background:**

Seizure duration in electroconvulsive therapy (ECT) is positively related with patients’ outcome. This study sought to investigate the impact of anesthetic management on seizure duration, and the impact of selected drugs (theophylline, remifentanil, S-ketamine) on seizure duration.

**Methods:**

Retrospective analysis of all patients undergoing ECT at our institution from January 2011 to April 2012 was performed based on electronic medical chart and review of existing quality improvement data. Patient data (*N* = 78), including gender, age, height, weight, and administered drugs, energy levels, and electroencephalic seizure duration were analyzed. Statistical analysis was performed using a generalized linear model.

**Results:**

A total of 78 patients (male = 39, female = 39, age 51 ± 12 years) were included. Average number of session was 10 ± 6 (1–30). In our patient population, theophylline administration was the only parameter, which significantly prolonged seizure duration, whereas S-ketamine, remifentanil, thiopental, age, sex, session or energy level had no significant effect.

**Conclusion:**

Theophylline can be a useful adjunct for patients with inadequate seizure duration. If there is a concomitant beneficial effect on patients’ outcome needs to be investigated in further studies.

## Background

Since the introduction of convulsive therapy by Ladislav Meduna in 1934 [1] and the first treatment in Germany performed at our institution in 1937 [2], major modifications have been made to this treatment in order to improve outcome and patients’ acceptance. Today electroconvulsive therapy (ECT) is one of the most effective treatments for patients with major depression and persistent psychosis [3] and is performed under general anesthesia with muscle relaxation. Since suicide is a leading cause of death among psychiatric patients, and also a leading cause of death from all causes in people younger than 30 years [4], many investigations to improve efficacy of ECT have been made in the last years [5].

Several studies thereby focused on drug management, for example S-ketamine was shown to lead to longer motor seizure duration than metohexital [6]. When etomidate was given for induction of anesthesia longer motor and electroencephalographic (EEG) seizures were observed compared to propofol [7]. Propofol dose dependently reduces seizure duration [8]. Aminophyllines like theophylline increase seizure duration [3].

Only a few studies focused on other parameters potentially affecting seizure quality for example demographic factors like age or gender, interventions to lower seizure threshold, or modifications to seizure induction such as stimulus duration or energy level. Older patients were shown to have relatively short (clonic phase) seizure duration [9], and an inverse relationship between ECT intensity and the clonic phase of induced seizure [9] was found.

Despite these investigations no standard therapy is currently considered to be the gold standard. Therefore authors routinely use a step up approach, which starts with barbiturates followed by etomidate, if the seizure duration with barbiturate induction is inadequate. If seizure duration is still too short with etomidate induction, other adjuncts, such as remifentanil, theophylline, S-ketamine, or a combination of these drugs is being used or the stimulation intensity is increased. The goal of this retrospective study was to investigate in a relatively large patient population which single action significantly prolonged seizure duration in patients undergoing ECT.

## Methods

### Study design

We retrospectively reviewed the medical charts of 78 patients who underwent a total of 786 ECT sessions. The patients were identified from the anesthesia electronical medical chart database (NarkoData, Hüttenberg, Germany). Other data (energy levels, EEG seizure duration) were derived from a quality improvement database at the psychiatry department. All patients were diagnosed with severe depression, which did not respond to pharmacological therapy. A psychiatrist made the decision for ECT. Patient data (including age, height, weight, and gender), administered drugs, energy levels, and seizure duration as measured by EEG were analyzed. We excluded patients with the need of bitemporal stimulation, patients who had to undergo a second therapy interval and patients that were taking medications that might alter seizure threshold such as benzodiazepines or bupropion.

The study was approved by the ethics committee of the University Erlangen-Nuremberg by short correspondence from May 2012 without the need of informed consent.

### Anesthesia

Anesthesia was performed as described previously [10]*.* In all patients standard monitoring, i.e. electrocardiography, automatic blood pressure measurement, pulse oximetry, and an intravenous catheter were established. Patients were preoxygenated with 100% oxygen by face mask for 3 min and either 5 mg/kg thiopental or 0.15 to 0.2 mg/kg etomidate was injected for induction of anesthesia. If necessary – see explanation below – application of remifentanil bolus of 0.5 μg/kg, 2 mg/kg theophylline or 0.3 mg/kg S-ketamine was added. After loss of consciousness 0.9 to 1.2 mg/kg succinylcholine was administered. Bag mask ventilation with 100% oxygen was performed by hyperventilating patients to achieve an end-tidal carbon dioxide of 27 to 30 mmHg. After relaxation a bite block was inserted and the electrical stimulus was applied in apnea by the psychiatrist. Energy level was chosen based upon the previous session by the anesthesiologist and psychiatrist. In the first treatment session energy level was set by titrating the individual seizure threshold.

After EEG seizure termination a bolus of 0.5 mg/kg propofol was injected to minimize risk of postictal agitation. Manually ventilation was again initiated until sufficient spontaneous breathing, i.e. patent airway without naso- or oropharyngeal airway and tidal volumes greater than 5 mL/kg body weight. Afterwards patients were transferred to the post anesthesia care unit where monitoring was continued until patients were brought back to the ward.

After each session the anesthesiologist and the psychiatrist decided depending on observed EEG seizure duration if there was a need to switch the induction agent from thiopental to etomidate or for the application of one or more additional drug(s), i. e. remifentanil, theophylline, or S-ketamine, or an increase of energy level for the next ECT session for the individual patient. Thereby only one parameter was changed or added for the next session.

### Electroconvulsive therapy

ECT was performed standardized in a series of single ECT sessions (2 or 3 times a week) under general anesthesia as described above. Electroencephalography was recorded continuously. Brief-pulse stimulation technique was applied with right unilateral stimulation using the Thymatron System II apparatus (Somatics, Lake Bluff, IL, USA).

Individual seizure threshold, defined as stimulus dosage that elicited an EEG seizure of at least 15 s was titrated during the first treatment. EEG seizure duration and quality were measured by the psychiatrist by analysis of spike and wave activity. Stimulus duration was in a range from six to eight seconds with a pulse width from 0.5 to 1.0 ms and a frequency from 10 to 70 Hz. Maximum output was 504 mC current.

### Statistics

Continuous interval scaled data were tested for normal distribution using the Shapiro-Wilk test. The influence of parameters and covariates on EEG seizure duration was analyzed using a generalized linear model for repeated measurements. The following parameters were included as nominal factors: sex, etomidate, S-ketamine, remifentanil, theophylline, thiopental, and session. Age and ECT energy were included as continuous covariates. Data are reported as mean ± SD and range. Statistical analysis was performed using IBM SPSS statistics 21.

## Results

We analyzed 786 ECT sessions of 78 patients. The demographic data are summarized in Table 1. Each patient underwent on average 10 ± 6 (1–30 ECT sessions. Mean EEG seizure duration was 58 ± 24 (3–172) seconds. The energy level ranged from 25.2 to 504 mC.

**Table 1:** Patient data. Data are given as mean ± SD (range) or number

Male/female	39/39
Age (yrs.)	51 ± 13 (21–77)
Body weight (kg)	83 ± 16 (42–135)
Height (cm)	172 ± 9 (155–192)
BMI (kg/m^2^)	28.1 ± 4.7 (15.2–42.6)

**Fig. 1:**
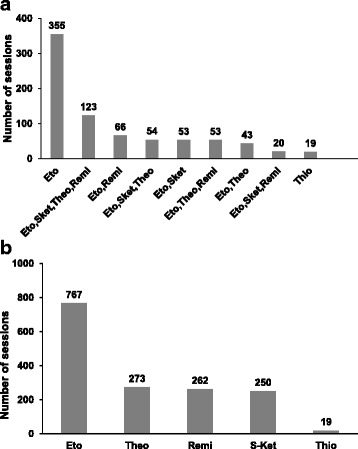
Incidence of administered drug combinations (**a**) and cumulative use of drugs (**b**). Eto: etomidate, Sket: S-ketamine, Theo: theophylline, Remi: remifentanil, Thio: thiopental

Figure 1a shows how often each drug was used over all ECT sessions. The three most common used drugs (combinations) were: etomidate alone, etomidate with S-ketamine, theophylline, and remifentanil, and etomidate with remifentanil. Figure 1b shows how often a drug was administered alone or a combination of one or more different drugs was used in the ECT sessions. Etomidate was the most commonly used drug, followed by theophylline, remifentanil and S-ketamine. These three drugs were used in comparable numbers of ECT sessions (Fig. 1b), which is important for the statistical power of this study. Thiopental did not play a substantial role. In about half of the ECT sessions etomidate was given alone. In the other half etomidate was combined with one or more other drugs.

Table 2 shows the results of the generalized linear model. Administration of theophylline was the only parameter with a significant effect on EEG seizure duration in our patient population. With theophylline, the estimated marginal mean (± standard error) of the EEG seizure duration was 64 ± 5 s, compared to 54 ± 5 s in sessions without theophylline (*p* < 0.001).

**Table 2:** Significance of the influence of the study parameters on EEG seizure duration, as obtained by analysis with a generalized linear model for repeated measurements

Parameter	Significance
Age	0.61
Sex	0.34
Etomidate	0.86
S-ketamine	0.58
Remifentanil	0.87
Theophylline	<0.001
Thiopental	0.98
Energy	0.076
Session	0.15

## Discussion

This study showed that in our patient population only theophylline significantly prolonged seizure duration whereas other drugs that are commonly used for ECT like S-ketamine, remifentanil, thiopental had no significant effect on EEG seizure duration. One has to keep in mind that the other interventions might still have an effect on seizure duration but have failed to reach statistical significance secondary to insufficient number of cases or smaller effect size. We also demonstrated that demographic factors such as age or gender and external factors such as number of sessions or energy level had – at least in our patient population - no significant effect on EEG seizure duration.

Despite the fact that several investigations on the influence of drugs on seizure quality and duration have been performed and published, there is no generally accepted treatment protocol.

Methohexital used to be the gold standard for ECT secondary to its minimal anticonvulsant effects compared to other barbiturates, but has fallen out of favour since there was a lack of availability especially on the US market. The introduction and widespread clinical use of other hypnotic drugs, such as etomidate and propofol also contributed to the decreased use of barbiturates. Tan et al. [7] performed a study in which 20 patients received either propofol for their first, and etomidate for their second ECT session or vice versa. They found that etomidate was associated with a significantly longer motor and EEG seizure duration compared to propofol. Vaidya et al. demonstrated in a retrospective study that patients receiving propofol required significantly more treatments than those receiving methohexital when right unilateral electrode placement was used. Propofol was also associated with a significantly higher requirement for bilateral ECT and higher stimulus dosing. Seizure duration was significantly shorter in the propofol condition, with more patients requiring re-stimulation for brief seizures [11]. In another study Sackeim et al. showed that increasing the electrical dosage increases the efficacy in patients receiving right unilateral ECT [12], which is known to have less cognitive side effects compared to bilateral stimulation.

A previous study reported that S-ketamine lead to longer motor seizure duration than metohexital [6]. However, in a recently published blinded study with more than 80 patients, no significant effect on seizure duration was found when ketamine was added to sevoflurane anesthesia for ECT [13]. Hooten demonstrated that combining methohexital or propofol with short-acting opioids such as remifentanil prolonged seizure duration in patients with inadequate seizure quality [14]. These findings have been corroborated by a recently published study that found longer seizure duration in patients receiving (a reduced dose of) propofol plus remifentanil when compared to propofol alone [15]. Our results suggest that adding of remifentanil has no significant effect on EEG seizure duration when etomidate is used for induction of anesthesia.

Theophylline is a nonselective adenosine receptor antagonist. Adenosine is a powerful endogenous anticonvulsant that can terminate seizures, and extracellular adenosine is increased after seizures in patients with intractable epilepsy [16]. Thus, theophylline with its adenosine antagonistic effects has been known to lower seizure thresholds and should be used judiciously in patients with pre-existing seizure disorders. In patients undergoing ECT with a history of inadequate seizure quality or duration this potential adverse effect can be used to improve and prolong seizure quality and duration, respectively.

Devanand et al. [17] described a case of a 23-year-old man undergoing ECT after a suicide attempt despite a 4-week treatment with haloperidol. The patient was also taking theophylline (1200 mg per day) for chronic asthma. The patient showed an extremely prolonged seizure that eventually stopped after receiving multiple boluses of diazepam for a cumulative dose of 20 mg. Swartz and Lewis [18] published an interesting study in which they used oral theophylline (200-400 mg) given 10 h prior to ECT in order to increase seizure length in patients that previously had insufficient seizure durations. They reported a significant average increase of almost 14 s and no adverse effects from theophylline. Leentjens et al. [19] administered theophylline intravenously 30 min prior to seizure induction to patients that developed seizure inhibition during a course of ECT. A dose of 100-200 mg was found to effectively prolong seizure durations in all patients with the same or lower electrical doses that had failed to elicit adequate seizures before. Similarly, Stern et al. [3] reported an increase in duration for both EEG and clinical seizure in patients receiving aminophylline. In addition, they showed that this effect was maintained for three subsequent ECT sessions. In a very recent retrospective study by Kemp et al. [20] the effect of orally administered theophylline on seizure duration was investigated. They found a significant increase in seizure duration after administering oral theophylline. We have chosen to administer theophylline intravenously for several reasons: 1) It is logistically easier to administer the drug a few minutes prior to seizure induction than to time the intake of the pill on the general psychiatric floor, 2) patients have intravenous access and standard monitors established and could be treated immediately, in the unlikely scenario of an adverse event such as tachycardia – and 3) intravenous administration is better accepted by patients.

Intravenously or orally administered caffeine has also been used to increase seizure duration [21]. Since intravenous caffeine is not readily available in the country where this study was performed and we did not want to rely on orally administered drugs for increasing seizure duration for the above mentioned reasons, we do not use this drug in our clinical practice.

Other demographic or clinical parameter like age, session number, or energy level did not reach statistical significance for an effect on the EEG seizure duration in our study. This is in contrast to a large study published by Rasimas et al. [22] where significant effects of both age and session number were reported. In this study however, the vast majority of the 519 patients received bitemporal stimulation, whereas we only included patients receiving unilateral stimulation. Similar to our results, gender did not significantly affect seizure duration in this study.

## Conclusion

In our study population only the effect of theophylline on EEG seizure duration in patient undergoing ECT reached statistical significance. Since prolonged seizure duration is supposed to be directly related with significant better outcome, theophylline may be effectively improving outcome in patients with short seizure duration. However, further investigations on correlation between drug management - especially use of theophylline - and patients’ outcome need to be performed.

## Abbreviations

ECT: Electroconvulsive therapy
EEG: Electroencephalogram
US: United States
